# Skeletal muscle proteomics links mitochondrial abundance with peak fat oxidation in physically active young males

**DOI:** 10.1113/JP290966

**Published:** 2026-05-28

**Authors:** Eloise K. Tarry, Shaun Mason, Ghazanfar Abbas Khan, Sofie G. Vestergaard, Emilie A. Petersen, Mike C. Olsen, Maria Hansen, Arthur Ingersen, Kirsten F. Howlett, Steen Larsen, Christopher S. Shaw, Jørn W. Helge

**Affiliations:** ^1^ Institute for Physical Activity and Nutrition (IPAN), School of Exercise and Nutrition Sciences Deakin University Geelong VIC Australia; ^2^ Department of Biomedical Sciences, Faculty of Health and Medical Sciences University of Copenhagen Copenhagen Denmark; ^3^ School of Life and Environmental Sciences & Centre for Sustainable Bioproducts Deakin University Geelong VIC Australia; ^4^ Nordic Bioscience Herlev Denmark; ^5^ Clinical Research Centre Medical University of Bialystok Bialystok Poland

**Keywords:** exercise, fat oxidation, metabolism, muscle, proteomics

## Abstract

**Abstract:**

The interindividual variability in peak fat oxidation (PFO) and the intensity at which this occurs (Fat_max_) has been attributed to physiological factors, diet and physical activity; however, few studies have examined the contribution of skeletal muscle characteristics. The present study examined the relationship between PFO, Fat_max_ and the skeletal muscle proteome in young, physically active males. Thirty‐four young, lean males were phenotyped through assessment of aerobic capacity, PFO, body composition, fasting blood samples and a muscle biopsy. Liquid chromatography mass spectrometry based proteomics was used to assess skeletal muscle protein abundance. Only absolute PFO (g min^−1^) was positively correlated with ${\dot{V}}_{O_{2}\mathrm{peak}}$ (*r* = 0.496, *P = *0.003). Few skeletal muscle proteins correlated with absolute PFO, whereas relative PFO and Fat_max_ were positively associated with numerous mitochondrial proteins enriched in metabolic pathways, oxidative phosphorylation and other mitochondrial processes. Mitochondrial proteome abundance was positively correlated with both relative PFO (*r* = 0.633, *P < *0.001) and Fat_max_ (*r* = 0.595, *P < *0.001). Mitochondrial complex‐specific analysis demonstrated that respiratory complex V was associated with both relative PFO and Fat_max_. Multiple regression analyses indicated that mitochondrial abundance and muscle glycogen explained 55% of the variability in relative PFO, whereas mitochondrial abundance alone explained 43% of the variability in Fat_max_. Absolute PFO was explained by a combination of ${\dot{V}}_{O_{2}\mathrm{peak}}$, mitochondrial abundance and muscle glycogen content (*r* ^2^ = 0.562). This untargeted proteomic approach highlights that skeletal muscle mitochondrial content contributes to the interindividual variability in PFO and Fat_max_ in lean, active young males.

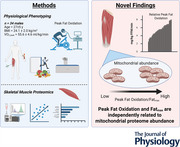

**Key points:**

- This study used an untargeted proteomics approach to explore the links between the skeletal muscle proteome and peak fat oxidation (PFO) in young, physically active males.
- Absolute PFO was primarily associated with maximal aerobic capacity. When expressed relative to fat‐free mass, PFO was closely associated with skeletal muscle proteins enriched in oxidative metabolism and mitochondrial pathways.
- Mitochondrial abundance assessed by mitochondrial proteome content and citrate synthase activity was positively related to relative PFO and the intensity at which this occurs (Fat_max_). Mitochondrial respiratory complex V was consistently related to both relative PFO and Fat_max_.
- Mitochondrial content independently contributed to both PFO and Fat_max_, highlighting mitochondrial content as a key determinant of the maximal capacity for fat oxidation.

## Graphical abstract

**Abstract figure legend** This study used a non‐targeted proteomic approach to explore the skeletal muscle determinants of peak fat oxidation (PFO) and the exercise intensity at which this occurs (Fat_max_). Comprehensive physiological phenotyping was completed in young, lean, physically active males, including blood and skeletal muscle sampling. Relative PFO and Fat_max_ were positively associated with numerous proteins enriched in mitochondrial pathways, including oxidative phosphorylation and other metabolic processes. Mitochondrial abundance assessed by mitochondrial protein content and citrate synthase activity positively correlated with relative PFO and Fat_max_. Mitochondrial complex‐specific analysis revealed respiratory complex V was most closely associated with these outcomes. These findings demonstrate that skeletal muscle mitochondrial abundance independently contributes to the variability in fat oxidation capacity during exercise in young, lean males.

## Introduction

Exercise intensity is a critical factor determining fuel selection by the working muscle. Although fatty acids are the predominant fuel at rest and during low to‐moderate intensity exercise, lipid utilisation declines in the transition from moderate to high intensity exercise, with a decline in the utilisation of both plasma‐derived fatty acids and intramuscular lipids (Romijn et al., 1993; van Loon et al., 2001). Peak fat oxidation (PFO) rates and the exercise intensity at which this is achieved (Fat_max_) can be systematically determined through an incremental exercise protocol first developed by Achten et al. (2002). This well established and widely used protocol has shown that PFO can be markedly influenced by physiological factors such as endurance capacity and training status, body composition, habitual diet, and biological sex (Chrzanowski‐Smith, Edinburgh, Thomas et al., 2021; Fletcher et al., 2017; Randell et al., 2017; Venables et al., 2005). Furthermore, PFO has been linked to metabolic health outcomes (Robinson et al., 2015; Rosenkilde et al., 2010), whereas the association between PFO and endurance performance is mixed (Frandsen et al., 2017; Maunder et al., 2022; Romer et al., 2020; Vest et al., 2018).

Previous work has shown substantial individual variability in PFO (Fletcher et al., 2017; Randell et al., 2017; Venables et al., 2005), with reported rates in the range 0.17–1.27 g min^−1^ across groups of athletes (Randell et al., 2017). The highest PFO rates (>1.5 g min^−1^) have been reported in athletes habitually consuming a ketogenic low carbohydrate diet (Volek et al., 2016). Several large‐scale studies have shown that ${\dot{V}}_{O_{2}\max}$, physical activity, body mass/composition and diet can collectively explain up to 50% of the variability in PFO when assessed at the whole‐body level (Fletcher et al., 2017; Randell et al., 2017; Venables et al., 2005), although up to 79% of this variability has been explained in a smaller study with duplicate measures of PFO (Chrzanowski‐Smith, Edinburgh, Thomas et al., 2021). When PFO is expressed relative to fat free mass, considerably less variability is explained by these common behavioural and physiological factors (Fletcher et al., 2017; Randell et al., 2017; Venables et al., 2005).

Given that skeletal muscle is the major site of fatty acid oxidation during exercise, several studies have examined the relationship between skeletal muscle characteristics and PFO (Chrzanowski‐Smith, Edinburgh, Smith et al., 2021; Dandanell et al., 2018; Maunder et al., 2022; Maunder et al., 2023; Nordby et al., 2006; Shaw et al., 2020; Stisen et al., 2006). This work has linked mitochondrial content, capillary density, muscle fibre type and various proteins involved in fatty acid transport, intramuscular lipid mobilisation and fatty acid oxidation with PFO. However, many of these studies are limited by the inclusion of relatively small cohorts, where associations are examined using pooled data from endurance‐trained and untrained individuals (Dandanell et al., 2018; Nordby et al., 2006; Shaw et al., 2020; Stisen et al., 2006). In several cases, associations between muscle‐specific factors and PFO are attenuated or absent when examined within separate groups. Examining associations across two disparate groups may increase the probability that observed associations are confounded by training status and related characteristics, including aerobic capacity, body composition and dietary factors and raise doubts about meaningful mechanistic relationships. Furthermore, studies to date have examined only a limited number of specific proteins considered important in fatty acid oxidation, lipid handling and aerobic metabolism (Chrzanowski‐Smith, Edinburgh, Smith et al., 2021; Maunder et al., 2022; Maunder et al., 2023; Nordby et al., 2006; Shaw et al., 2020).

Mass spectrometry (MS) based proteomics offers an opportunity to more broadly examine skeletal muscle protein composition. Recent human metabolic research studies have utilised proteomic approaches combined with in‐depth metabolic phenotyping to link individual variability in metabolic/physiological traits with the muscle proteome (Emanuelsson et al., 2024; Kjaergaard et al., 2025; Needham et al., 2024). However, to date, these non‐targeted approaches have not been used to determine whether the skeletal muscle proteome is related to the individual variability in PFO. The present study aimed to utilise global MS based proteomics combined with comprehensive metabolic phenotyping to examine the relationship between the skeletal muscle proteome and PFO in young, physically active males.

## Methods

### Participants

**Figure 1:**
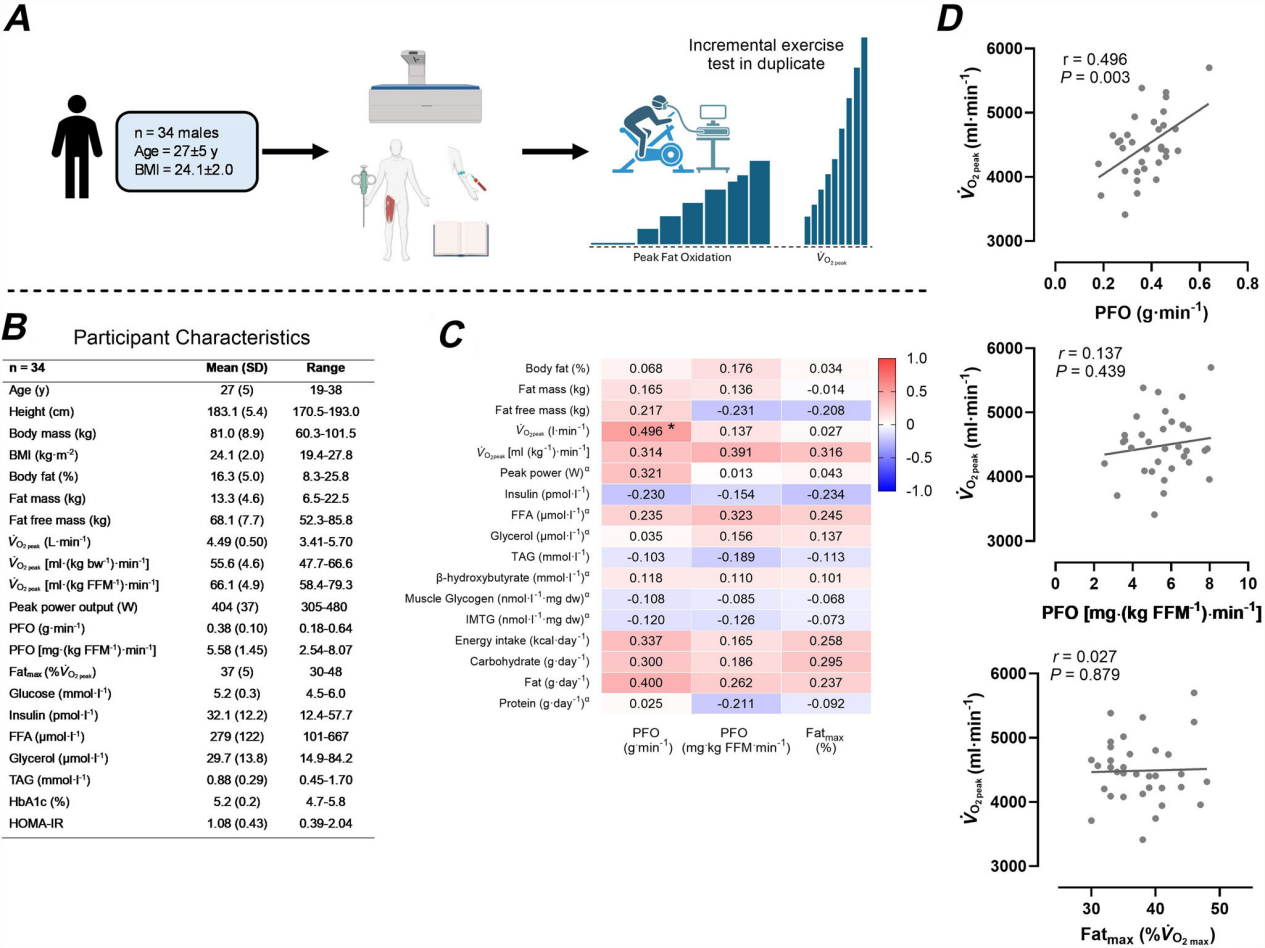
Physiological and metabolic phenotyping *A*, flowchart of study design depicting metabolic phenotyping and blood and skeletal muscle sampling. *B*, participant characteristics. *C*, heatmap showing associations (Pearson's correlations) between absolute and relative PFO and Fat_max_ with physiological characteristics (^α^Spearman's rank associations). *Significant correlation following adjustment for false discovery rate. *D*, associations between absolute ${\dot{V}}_{O_{2}\mathrm{peak}}$ with absolute and relative PFO and Fat_max_.

The study conformed to the standards set by the Declaration of Helsinki (2013) and was registered with The Danish Data Protection Agency (8514‐0549/20‐3000). The study was approved by the Science Ethical Committee of the greater region of Copenhagen, Denmark (H‐20019103) and by the Deakin University Human Research Ethics Committee (2021‐047). Thirty‐four moderately to well‐trained males participated in this study (Fig. 1). Inclusion criteria included those completing moderate intensity endurance exercise ≥3 times per week, ${\dot{V}}_{O_{2}\mathrm{peak}}$ ≥45 mL kg^−1^ min^−1^, body mass index <27 kg m^−2^ and aged 18–40 years. All participants were free of metabolic or cardiovascular disease and of any known contraindication to exercise. Written informed consent was obtained prior to participation in the study. This study included a subset of participants from a previously published study (Tarry et al., 2025).

### Physiological phenotyping

#### Visit 1

Participants were initially screened for eligibility and completed the International Physical Activity Questionnaire (i.e. IPAQ) (Craig et al., 2003)). Participants were asked to refrain from vigorous exercise, consume their habitual diet at the same time as avoiding alcohol and caffeine for 24 h prior to the first visit. Following an overnight fast of ≥9 h, participants were advised to travel by public transport or car and arrived at the laboratory between 07.00 h and 09.00 h. An incremental exercise test on a cycle ergometer (Ergomedic 839E; Monark, Vansbro, Sweden) was completed to determine rates of peak fat oxidation (PFO), the exercise intensity at which PFO occurs (Fat_max_) and ${\dot{V}}_{O_{2}\mathrm{peak}}$. This test has been previously described by our group (Frandsen et al., 2019). Briefly, the test began with 5 min of seated rest on the cycle ergometer, followed by a 5 min warm‐up at 60 W. The workload was then increased by 35 W every third minute until the respiratory exchange ratio exceeded 1.0. Following a 5 min rest period, participants underwent an incremental exercise test to exhaustion to determine ${\dot{V}}_{O_{2}\mathrm{peak}}$. Following commencement of the test at 95 W, the workload increased by 35 W min^−1^, until exhaustion. Pulmonary ${\dot{V}}_{O_{2}}$ and ${\dot{V}}_{{\mathrm{CO}}_{2}}$ were measured breath‐by‐breath at 10‐s intervals using indirect calorimetry (Quark CPET; Cosmed, Rome, Italy). Substrate oxidation was calculated from the last 90 s of each stage of the PFO test with the assumption that the urinary nitrogen excretion rate was negligible (Frayn, 1983). Fat oxidation rates were plotted against the %${\dot{V}}_{O_{2}\mathrm{peak}}$, and PFO and Fat_max_ were determined using second degree polynomial regression analysis, comprising a widely adopted curve‐fitting approach in this field (Amaro‐Gahete et al., 2019). The screening visit formed the first of two peak fat oxidation tests, and PFO and Fat_max_ are reported as the average of the two tests, as recommended given the day‐to‐day variability in measures of PFO (Chrzanowski‐Smith et al., 2020; Croci et al., 2014).

#### Visit 2

Within 4 weeks of visit 1, participants followed identical pre‐visit dietary and exercise requirements described for visit 1 and again arrived at the laboratory following an overnight fast of >9 h. Dual‐energy X‐ray absorptiometry (Lunar Prodigy Advance; Lunar, Madison, WI, USA) was performed to determine body composition. Body mass and height were assessed for body mass index calculation.

Resting blood samples (50 mL) were collected from the antecubital vein using vacutainer tubes with anticoagulants [dipotassium (K2) ethylenediaminetetraacetic acid (EDTA)/aprotinin (K2EDTA), tripotassium (K3) EDTA/aprotinin (250 U of aprotinin and K3E15%) and lithium heparin (LH 34 IU)] and immediately centrifuged at 2800 *g* at 4°C for 10 min. A muscle biopsy was then obtained under local anaesthesia (5 mL of xylocaine 20 mg mL^−1^) from the musculus vastus lateralis, using a modified Bergström technique with applied suction. Tissue samples were collected (∼100 mg), visible blood and connective tissue was cleaned, and samples were frozen immediately in liquid nitrogen. Plasma and muscle samples were stored at −80°C for later analysis. After a 30 min recovery period, participants completed another incremental exercise test for the determination of PFO and Fat_max_, as described for visit 1.

### Physical activity and habitual diet registration

Prior to visit 2, 4 day weighed online dietary records were collected to evaluate habitual dietary intake across consecutive days, including one weekend day (MADLOG ApS, Kolding, Denmark). Over the same period, habitual physical activity and energy expenditure were monitored for 4 days using an activity watch (Fitbit Charge 4; Fitbit LLC, San Francisco, CA, USA), estimating total daily energy expenditure by integrating participant characteristics (age, sex, height and weight) with heart rate and accelerometer‐derived activity data.

### Skeletal muscle analysis

A portion of skeletal muscle was freeze‐dried and connective tissue, visible fat and blood were dissected using a stereomicroscope. Maximal citrate synthase (CS) enzyme activity, in addition to intramuscular triglyceride (IMTG) and muscle glycogen content, were analysed as previously described (Frandsen et al., 2022).

### MS

#### Sample preparation

Freeze‐dried powdered muscle samples (1–2 mg) were lysed in a buffer containing 150 mm HEPES (pH 7.3), 1 mm EDTA, 0.1 mm neocuproine and 2% SDS with a needle sonicator (Vibra‐Cell; Sonics and Materials, Inc., Newtown, CT, USA) for 6 × 10 s at amplitude 30. Samples were pre‐cleared by centrifugation at 17,500 *g* for 10 min at 4°C, followed by precipitation with 1 ml ice cold acetone for 1h at −20°C. Samples were then centrifuged at 4500 ***g*** for 20 min at 4°C and the supernatant removed. Pellets were washed twice with ice cold acetone, and then finally resuspended in a buffer containing 50 mm ammonium bicarbonate (pH 8.0), 1 mm EDTA, 0.1 mm neocuproine and 2% SDS. The protein concentration was measured using a BCA assay, and 10 µg of protein was used in sample preparation. Samples were diluted to 90 µL with 100 mm HEPES and 1% SDS, vortexed and then heated at 75°C for 10 min. After briefly cooling at room temperature, samples were simultaneously reduced and alkylated with 9 mm Tris(2‐carboxyethyl)phosphine hydrochloride and 40 mm iodoacetamide, respectively, with brief vortexing and heating at 95°C for 5 min. After cooling at room temperature, 0.2 mg of washed Sera‐Mag Carboxylate‐Modified Magnetic Beads (Cytivia, Marlborough, MA, USA) were combined with the sample and 120 µL of ethanol, and then shaken at room temperature for 8 min using a Thermomixer (Eppendorf, Hamburg, Germany) at 1600 rpm. Samples were placed on a magnetic bead rack and the lysis was discarded, followed by 3 × 1 mL washes with 80% (v/v) ethanol (Hughes et al., 2019).

Samples were resuspended in 50 µL of digestion buffer, containing 10% (v/v) trifluoroethanol in 100 mm HEPES, pH 7.5, and shaken for 2 min using a Thermomixer at 1600 rpm for 2 min. Trypsin at a ratio of 100:1 (protein:trypsin) was added to samples. Samples were then shaken at 1200 rpm using a Thermomixer for 18 h overnight at 37°C. After digestion, samples were placed on a magnetic rack and the sample supernatant taken. Two‐layered styrenedivinylbenzene‐reverse phase sulfonate stage tips were prepared using 0.2 mL Eppendorf pipette tips. Tips were equilibrated sequentially with 50 µL acetonitrile, 50 µL of 30% methanol with 0.2% trifluoroacetic acid (TFA), and 50 µL 0.2% TFA. Samples, along with 150 µL of 1% TFA were loaded onto tips and centrifuged at 500 ***g*** for 3 min, followed by sequential washes with 0.2% TFA (× 2) and 90% isopropanol with 1% TFA. Samples were eluted with 60 µL (v/v) of 80% acetonitrile/5% ammonium hydroxide and then vacuum dried in a SpeedVac (Thermo Fisher Scientific, Waltham, MA, USA) at 45°C. Samples were finally resuspended in 20 µL of buffer containing 2% acetonitrile and 0.1% TFA prior to liquid chromatography (LC)/MS analysis.

#### LC/MS

Samples were analysed using an UltiMate 3000 RSLCnano system (Thermo Fisher Scientific) interfaced with an Orbitrap Exploris 240 Mass Spectrometer (Thermo Fisher Scientific). Peptide samples (2 µL) were loaded onto a C18 nanoViper trap column for 3 min at a flow rate of 15 µL min^−1^ using 2% acetonitrile and 0.1% TFA, and then separated on a PepMap Neo C18 nanoViper analytical column (2 µm × 75 µm × 500 mm) at a flow rate of 250 nL min^−1^. Buffer A included 0.1% formic acid and Buffer B consisted of 80% acetonitrile with 0.1% formic acid. Gradient conditions were as follows: 0–2 min 2.5% B, 2–2.1 min 8% B, 2.1–122 min 32% B, 122–125 min 45% B, 125–133 min 99% B, and 133–150 min 2.5% B. The column compartment temperature was maintained at 50°C.

Positive ion voltage was set at 2000 V, and the ion transport tube temperature was set at 275°C. Data acquisition occurred in data‐dependent mode with 3 s between master scans. Full scan MS1 resolution was 120,000 (at *m*/*z* 200), with scan range *m*/*z* 350–1200. MS2 resolution was 15,000 using a normalised collision energy of 30% for higher‐energy collisional dissociation. Data were acquired using Xcalibur software (Thermo Fisher Scientific).

#### Data analysis

Raw data files were analysed using Proteome Discoverer, version 2.4 (Thermo Fisher Scientific). The Sequest HT algorithm was used with parameters including a maximum of two missed tryptic cleavages, a precursor mass tolerance of 10 ppm and fragment mass tolerance of 0.02 Da. Dynamic modifications were oxidation (methionines) and static modifications were carbamidomethylation (on cysteines). The Percolator node was used in the processing, with a strict target false discovery rate (FDR) set at 0.01 and relaxed FDR set at 0.05. The target/decoy strategy was concatenated with validation based on the *q* value. Normalisation of data was based on total peptide amount. For quantification, protein abundances were calculated on the basis of summed peptide abundances of unique and razor peptides. In subsequent statistical analyses, only identified master proteins with a 1% FDR were considered. The mass spectrometry proteomics data have been deposited in the ProteomeXchange Consortium via the PRIDE (Perez‐Riverol et al., 2025) partner repository (dataset identifier: PXD064359 and https://doi.org/10.6019/PXD064359).

#### Statistical analysis

Data were analysed in Prism (GraphPad Software Inc., San Diego, CA, USA) and SPSS (IBM Corp., Armonk, NY, USA). All variables were assessed for normality using the Shapiro–Wilk test. Correlations between metabolic outcomes (PFO and Fat_max_) and physiological variables (${\dot{V}}_{O_{2}\mathrm{peak}}$, CS activity, IMTG and glycogen) were examined using Pearson's correlation for normally distributed variables and Spearman's rank correlation for non‐normally distributed variables. Correlations between the skeletal muscle proteome and metabolic outcomes (PFO and Fat_max_) were examined using Spearman's rank correlation. All *P* values from correlation analysis were corrected using the Benjamini–Hochberg FDR procedure. Proteins significantly associated with each metabolic outcome (FDR‐adjusted *P < *0.05) were taken forward for functional interpretation. Functional enrichment analysis for these significantly associated proteins was performed as an over‐representation analysis of Gene Ontology (GO) (http://geneontology.org) Biological Process terms via the clusterProfiler (https://bioconductor.org/packages//release/bioc/html/clusterProfiler.html) and https://bioconductor.org/packages//release/data/annotation/html/org.Hs.eg.db.html packages in R (R Foundation, Vienna, Austria), using gene symbols as identifiers and the full set of proteins tested in the correlation analysis as the background universe; enriched categories were adjusted using Benjamini–Hochberg correction and redundant terms were reduced using semantic similarity filtering prior to visualisation. Protein interaction networks for proteins positively associated with relative PFO were generated using STRING (https://cn.string‐db.org), with confidence network edges restricted to high‐confidence interactions (minimum interaction score 0.700). Mitochondrial proteins were identified based on Gene Ontology annotation (GO: 0005739). To estimate mitochondrial protein abundance, raw intensities for mitochondrial proteins were summed for each sample and expressed relative to the summed raw intensities across all quantified proteins to derive a mitochondrial fraction. For mitochondrial respiratory complex analyses, proteins were assigned to OXPHOS complexes I–V using a curated complex mapping table (see Supporting information, Table S1) and, within the mitochondrial protein set, raw intensities were summed per complex and expressed as a percentage of the total mitochondrial proteome (complex fraction = 100 × complex sum/mitochondrial sum). Associations between mitochondrial complex fractions and metabolic outcomes were assessed using Kendall's rank correlation (τ). Stepwise multiple regression analysis was performed to examine the independent influence of physiological and muscle characteristics on absolute PFO, relative PFO and Fat_max_. Potential independent variables were pre‐screened for collinearity using bivariate correlations, with removal of one variable in the case of strong associations (*R* > 0.8). *P < *0.05 was considered statistically significant. All data are reported as the mean ± SD, unless otherwise stated.

## Results

### Physiological and metabolic phenotyping

A summary of the study design and participant characteristics are presented in Fig. 1*A* and *B*. In total, 34 males met the inclusion criteria and completed the physiological testing and sample collection. The participants were young (27 ± 5 years, range 19–38 years), lean (16.3 ± 5.0% body fat, range 8.3–25.8% body fat) and had a high ${\dot{V}}_{O_{2}\mathrm{peak}}$ (55.6 ± 4.6 mL kg^−1^ min^−1^, range 47.7–66.6 mL kg^−1^ min^−1^). All participants consumed a typical balanced diet with reported energy intake of 3122 ± 624 kcal with proportions from carbohydrate, fat and protein of 48.4 ± 4.4%, 31.7 ± 5.5% and 17.2 ± 3.7%, respectively. Energy expenditure was estimated to be 3545 ± 546 kcal/day.

The average PFO of two separate visits are presented in Fig. 1*B*. Absolute PFO averaged 0.38 ± 0.10 g min^−1^ and relative PFO averaged 5.58 ± 1.45 mg kg fat‐free mass (FFM)^−1^ min^−1^. The coefficient of variation was 23% for PFO and 8% for Fat_max_, which aligns with previous reports (Chrzanowski‐Smith et al., 2020). There was no difference in average PFO between visit 1 (0.37 ± 0·11 g min^−1^, 5.47 ± 1.78 mg kg FFM^−1^ min^−1^) *vs*. visit 2 (0.39 ± 0.14 g min^−1^, 5.69 ± 2.00 mg kg FFM^−1^ min^−1^). Despite a relatively homogenous group of young, moderately‐well trained, lean males, there was substantial interindividual variation in PFO which varied 3.5‐fold (range 0.18–0.64 g min^−1^) and 3‐fold (range 2.54–8.07 mg kg FFM^−1^ min^−1^) for absolute and relative PFO, respectively (Fig. 1*B*). PFO occurred at an average of 37 ± 5% ${\dot{V}}_{O_{2}\mathrm{peak}}$ (range 30–48%) and this did not differ between visits 1 and 2 (38 ± 5% *vs*. 37 ± 6% ${\dot{V}}_{O_{2}\mathrm{peak}}$). Associations between PFO and Fat_max_ with physiological outcomes are shown in Fig. 1*C* and *D*. Absolute PFO was positively associated only with absolute ${\dot{V}}_{O_{2}\mathrm{peak}}$ (*r* = 0.496, *P = *0.003). Relative PFO and Fat_max_ were not associated with any physiological outcomes following adjustment for false discovery rate.

### Skeletal muscle proteomic signatures of absolute PFO

**Figure 2:**
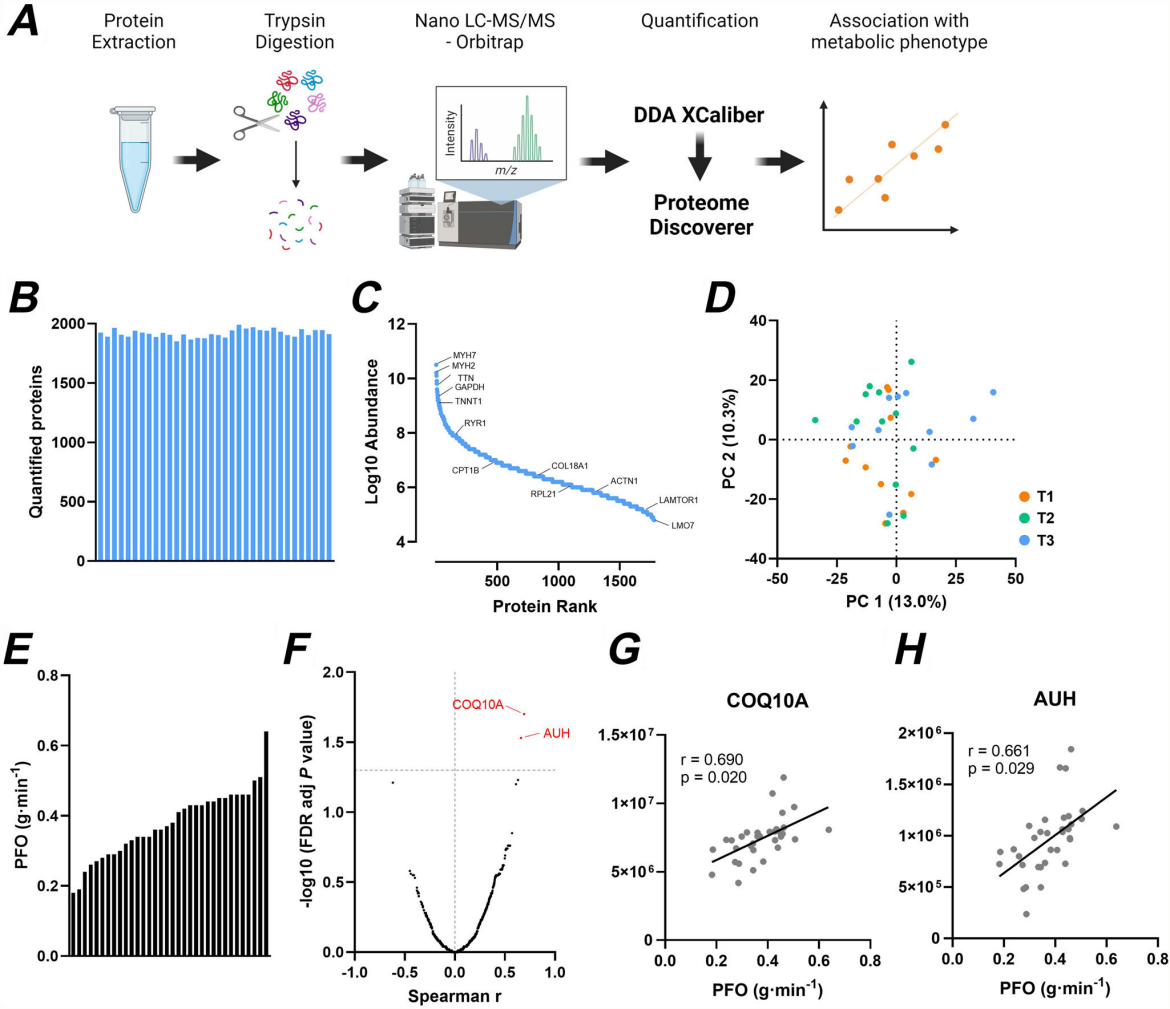
Skeletal muscle proteome and associations with PFO (g min^−1^) *A*, flowchart of proteomics workflow using nano LC‐MS/MS Orbitrap. Samples were measured in data dependent acquisition mode and quantified using Proteome Discoverer software. *B*, number of quantified proteins per sample. *C*, ranked abundances of proteins in skeletal muscle. *D*, principal component (PC) analysis of the proteome of skeletal muscle samples, coloured by tertile of absolute peak fat oxidation rate (g min^−1^). *E*, ranked bar plot demonstrating absolute PFO (g min^−1^) heterogeneity across all individuals. *F*, volcano plot showing Spearman's rank correlation coefficients (*r*) with PFO (g min^−1^), significant proteins following Benjamini–Hochberg correction for multiple analyses highlighted in red. *G* and *H*, significant associations between individual protein abundance, coenzyme Q‐binding protein COQ10 homolog A (COQ10A) (*G*) and methylglutaconyl‐CoA hydratase (AUH) (*H*) and absolute PFO (Spearman's rank).

Proteomic analysis was performed on skeletal muscle biopsies collected in the overnight fasted and rested state to assess the relationship between the skeletal muscle proteome and PFO (Fig. 2*A*). A summary of quantified proteins is shown in Fig. 2*B*. In total, 2325 proteins were detected, with 90% of these proteins detected in 70% of the samples. Proteins involved in muscle contraction, excitation–contraction coupling, metabolic enzymes, and mitochondrial proteins were highly abundant in the muscle samples (Fig. 2*C*). Principle component analysis is shown in Fig. 2*D*, separated by tertiles of PFO (g min^−1^).

To explore relationships between the skeletal muscle proteome and individual variability in PFO (Fig. 2*E*), we examined associations between absolute PFO (g min^−1^) and skeletal muscle proteins identified with an FDR of <1% (see Supporting information, Table S2). Following corrections for multiple analyses, two proteins were positively associated with absolute PFO [coenzyme Q‐binding protein COQ10 homolog A (COQ10A) and methylglutaconyl‐CoA hydratase (AUH)] (Fig. 2*F*–*H*). These proteins have functions in distinct processes. COQ10A is required for the function of coenzyme Q in the respiratory chain and AUH is involved in the degradation of leucine. However, it is notable that these proteins are localised to the mitochondria.

### Skeletal muscle proteomic signatures of relative PFO

**Figure 3:**
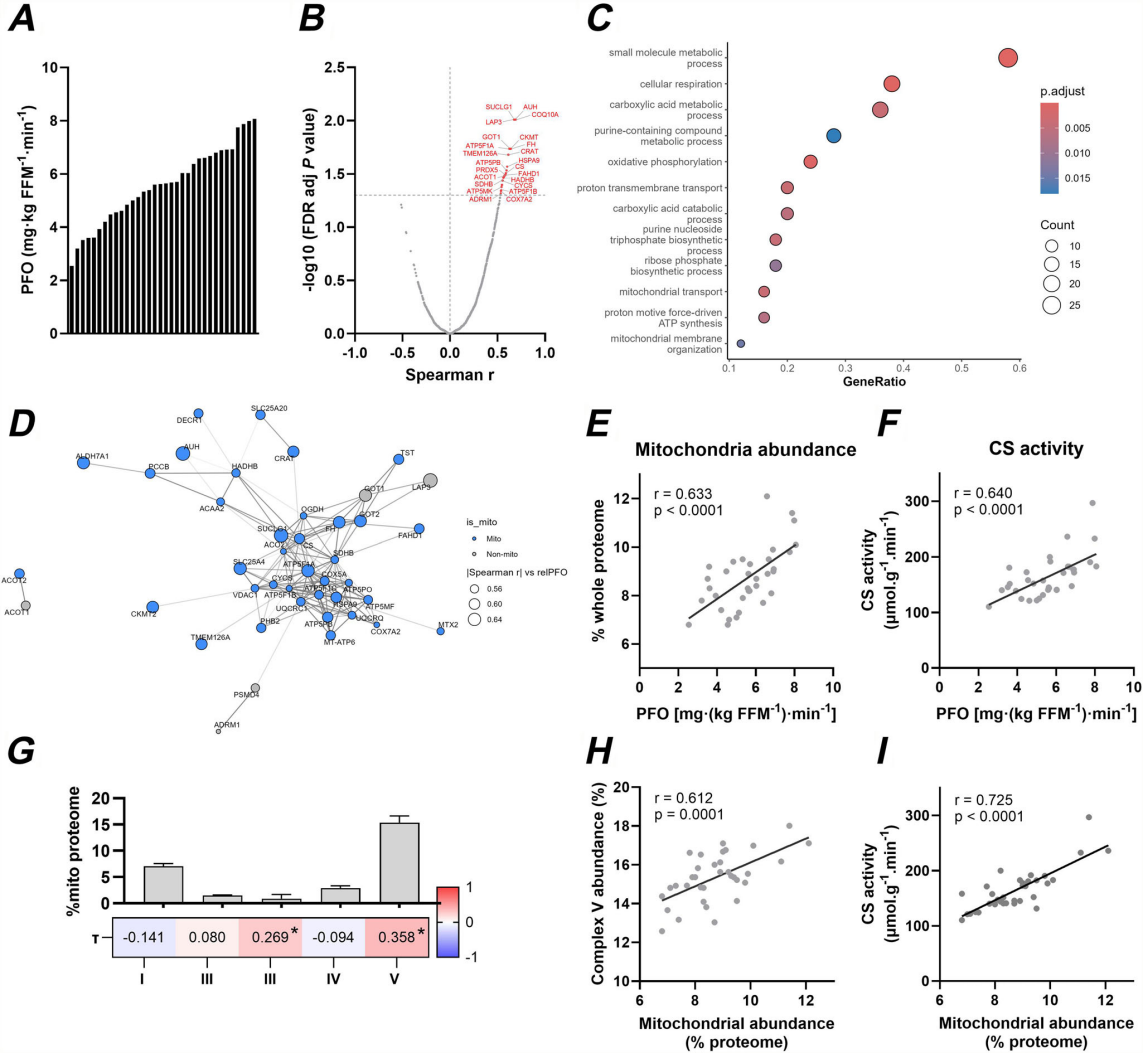
Skeletal muscle proteome and associations with relative PFO (mg kg FFM^−1^ min^−1^) *A*, ranked bar plot demonstrating relative PFO (mg kg FFM^−1^ min^−1^) heterogeneity across all individuals. *B*, volcano plot showing Spearman's rank correlation coefficients (*r*) with PFO, significant proteins highlighted in red following Benjamini–Hochberg correction for multiple analyses. *C*, pathway enrichment analysis (GO Biological Process) based on significant positive Spearman's rank correlations with PFO (Benjamini–Hochberg FDR‐adjusted *P < *0.05). *D*, protein–protein interaction network of proteins significantly and positively associated with relative PFO. Interactions were obtained using STRING with high‐confidence edges (minimum interaction score 0.700). Nodes are coloured by subcellular localisation: mitochondrial (blue) and non‐mitochondrial (grey). *E* and *F*, associations (Spearman's rank) between relative PFO and mitochondrial content as estimated through mitochondrial proteome abundance (*E*) and CS activity (*F*)*. G*, Kendall's rank coefficient (τ) of individual mitochondrial complex abundance (expressed as a percentage of total mitochondrial proteome) and relative PFO. *H*, correlations between mitochondrial abundance and mitochondrial complex V abundance. *I*, Pearson's correlations between CS activity and mitochondrial proteome abundance.

We next examined relationships between the skeletal muscle proteome and relative PFO (corrected for fat‐free mass) (Fig. 3*A*). Following correction for multiple analyses, there were 51 proteins that positively correlated with relative PFO (Fig. 3*B*; see also Supporting information, Table S2). These included the two proteins that positively correlated with absolute PFO (COQ10A and AUH). Enrichment analysis was performed for positively associated proteins based on GO Biological Process databases using Clusterprofiler. Proteins were highly enriched in metabolic pathways (FH, CRAT, CS, PCCB, ACOT1 and HADHB), cellular respiration (ATP5F1A, ATP5PB, ATP5MF COX5A, COX7A2, CYCS and SDHB) and mitochondrial specific transport and organisation (SLC25A4, VDAC1, MTCH2, TMEM126A and HSPA9) (Fig. 3*C*). The majority of the positively associated proteins were localised to the mitochondria (Fig. 3*D*). Proteins that did not localise to the mitochondria were cytosolic proteins involved in amino acid metabolism (LAP3 and GOT1), fatty acid handling (ACOT1) and ubiquitin receptors within the 26S proteasome (PSMD4 and ADRM1).

Considering the proteins positively associated with relative PFO were highly enriched for mitochondrial proteins, we examined the relationship between relative PFO and mitochondrial proteome abundance. Mitochondrial proteome abundance was highly positively correlated with relative PFO (*r* = 0.633, *P < *0.0001) (Fig. 3*E*). Similar positive associations were also observed between relative PFO and skeletal muscle CS activity (*r* = 0.640, *P < *0.0001) (Fig. 3*F*). We further examined the electron transport chain complexes and found that the ATP‐synthase complex (complex V) most strongly correlated with relative PFO (τ = 0.358, *P = *0.003) (Fig. 3*G*), which was also the complex with the strongest association with mitochondrial proteome abundance (*r* = 0.612, *P = *0.0001) (Fig. 3*H*) and CS activity (*r* = 0.601, *P = *0.0002) (data not shown). Finally, we also examined the correlation between CS activity and the mitochondrial proteome abundance, which were highly correlated (*r* = 0.725, *P < *0.0001) (Fig. 3*I*) demonstrating the validity of the mitochondrial proteome estimation of mitochondrial abundance.

### Skeletal muscle proteomic signatures of Fat_max_

**Figure 4:**
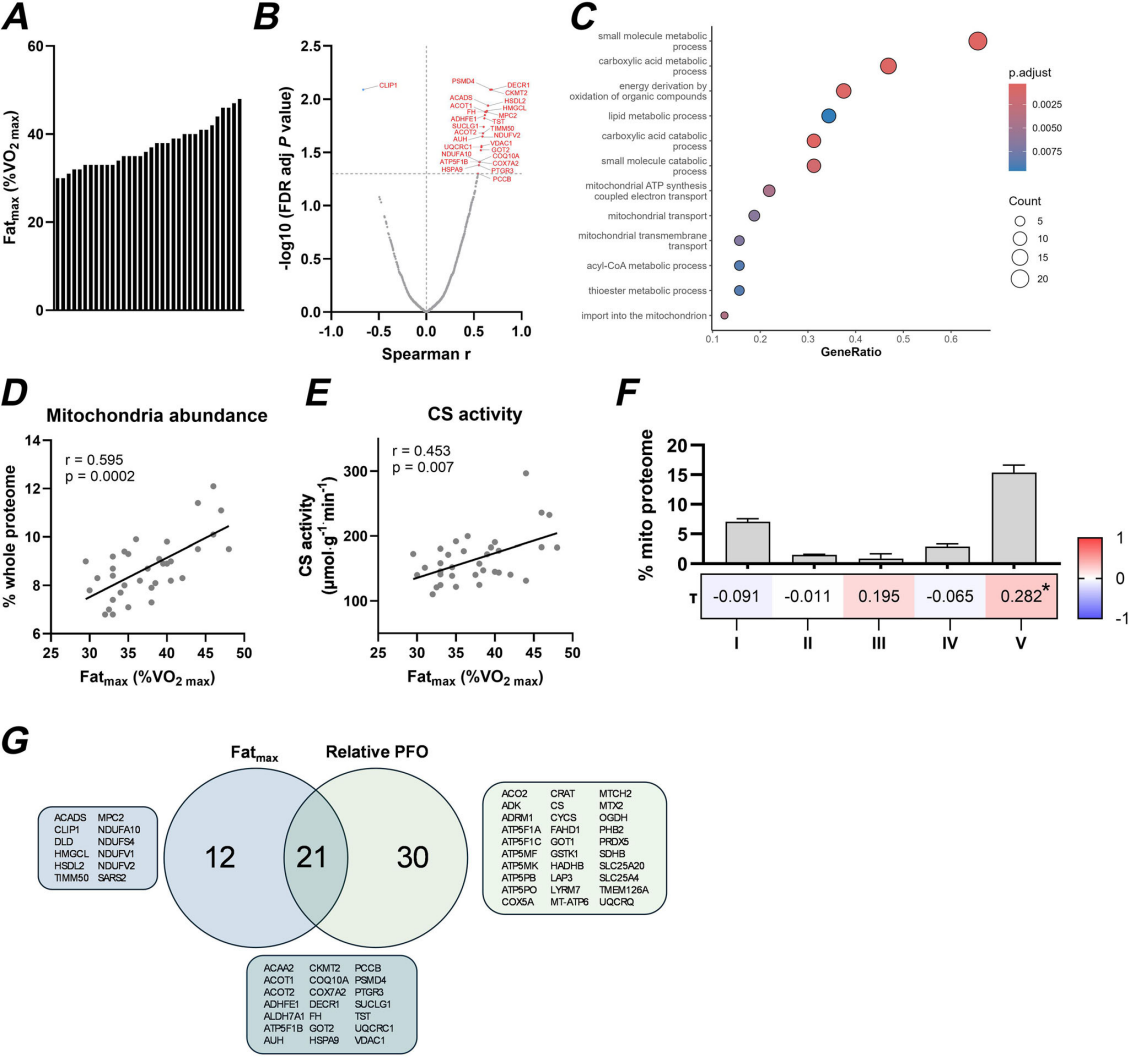
Skeletal muscle proteome and associations with Fat_max_ (%${\dot{V}}_{O_{2}\mathrm{peak}}$) *A*, ranked bar plot demonstrating Fat_max_ (%${\dot{V}}_{O_{2}\mathrm{peak}}$) heterogeneity across all individuals. *B*, volcano plot showing Spearman's rank correlation coefficients (*r*) with Fat_max_, with significant proteins highlighted in red (positive association) or blue (negative association) following Benjamini–Hochberg correction for multiple analyses. *C*, pathway enrichment analysis (GO Biological Process) based on significant positive Spearman's rank correlations with Fat_max_ (Benjamini–Hochberg FDR‐adjusted *P < *0.05). *D* and *E*, associations (Spearman's rank) between Fat_max_ and mitochondrial content as estimated through mitochondrial proteome abundance (*D*) and CS activity (*E*). *F*, Kendall's rank coefficient (τ) of mitochondrial complex abundance (% of total mitochondrial proteome) and Fat_max_. *G*, Venn diagram showing identified proteins that are significantly correlated with relative Fat_max_ and relative PFO.

We also examined associations between the skeletal muscle proteome and Fat_max_. Following correction for multiple analyses, 33 proteins were identified that significantly correlated with Fat_max_ (Fig. 4*B*; see also Supporting information, Table S2). Of these proteins, 32 positively associated and one negatively associated with Fat_max_. CAP‐Gly domain containing linker protein 1 (CLIP1), which is involved in regulating microtubule cytoskeleton dynamics, was the only protein negatively associated with Fat_max_.

Enrichment analysis demonstrated that positively associated proteins were enriched in metabolic pathways, lipid metabolism (AUH, ACOT1, ACOT2, DECR1, ACAA2 and ACADS) and mitochondrial‐related process including ATP synthesis coupled to electron transport (NDUFV2, NDUFV1, UQCRC1 and COX7A2) and mitochondrial transmembrane transport and import (HSPA9, VDAC1, TIMM50 and MPC2) (Fig. 4*C*). The majority of these proteins (29 of 32 proteins) were localised to the mitochondria. Mitochondrial content estimated from the mitochondrial proteome abundance (*r* = 0.595, *P = *0.0002) (Fig. 4*D*) and CS activity (*r* = 0.453, *P = *0.007) (Fig. 4*E*) were also positively correlated with relative Fat_max._ When exploring the electron transport chain complexes, the ATP‐synthase complex (complex V) positively correlated with Fat_max_ (τ = 0.282, *P = *0.020) (Fig. 4*F*). The majority of those proteins associated with Fat_max_ (21 of the 33 proteins identified) were also associated with relative PFO (Fig. 4*G*). Almost all of these were proteins regulating metabolism and localised to the mitochondria, with around half of these proteins involved in lipid metabolism.

### Multiple regression analysis to explain the variability in PFO

To examine the independent influence of muscle‐specific variables on PFO and Fat_max_, mitochondrial protein abundance and muscle glycogen were chosen as muscle‐related independent variables. This was based on the positive correlations between mitochondrial abundance and both PFO and Fat_max_ in this study, and the well described impact of muscle glycogen content on substrate utilisation during exercise reported previously (Gollnick et al., 1981; Wojtaszewski et al., 2003). These were included in a stepwise multiple linear regression alongside absolute ${\dot{V}}_{O_{2}\mathrm{peak}}$, as the most widely reported physiological determinant of PFO. Absolute ${\dot{V}}_{O_{2}\mathrm{peak}}$, mitochondrial proteome abundance and muscle glycogen content made significant independent contributions to absolute PFO, collectively explaining 56% of the variation in PFO (Table 1). Mitochondrial proteome abundance and muscle glycogen content were independently associated with relative PFO, explaining 55% of the variability, with no independent association with ${\dot{V}}_{O_{2}\mathrm{peak}}$ (Table 1). In terms of Fat_max_, mitochondrial proteome abundance explained 43% of the variability in Fat_max_ with no independent associations with either ${\dot{V}}_{O_{2}\mathrm{peak}}$ or muscle glycogen content (Table 1).

**Table 1:** Stepwise multiple linear regression for PFO and Fat_max_

	Standardised coefficient	*P* value	*r* ^2^	Adjusted *r* ^2^
**Absolute PFO (g min^−1^), *n* = 34**			0.562	0.518
Mitochondrial proteome (%)	0.549	<0.001		
${\dot{V}}_{O_{2}\mathrm{peak}}$ (L min^−1^)	0.443	0.001		
Muscle glycogen	−0.391	0.005		
**Relative PFO (mg kg^−1^ min^−1^), *n* = 34**			0.549	0.520
Mitochondrial proteome (%)	0.761	<0.001		
Muscle glycogen	−0.407	0.003		
**Fat_max_ (%** ${\dot{V}}_{O_{2}\mathrm{peak}}$ **), *n* = 34**			0.427	0.409
Mitochondrial proteome (%)	0.654	<0.001		

*Note*: Independent variables used in the model were ${\dot{V}}_{O_{2}\mathrm{peak}}$ (L min^−1^), mitochondrial proteome abundance and muscle glycogen concentration.

Abbreviation: PFO, peak fat oxidation rate.

## Discussion

The substantial individual variability in PFO has been partly explained by physical activity, diet and physiological factors, including aerobic capacity and skeletal muscle characteristics (Dandanell et al., 2018; Fletcher et al., 2017; Nordby et al., 2006; Randell et al., 2017; Shaw et al., 2020; Venables et al., 2005). In the present study, we utilised MS based proteomics to examine the relationship between PFO and Fat_max_ and the skeletal muscle proteome. Numerous mitochondria‐associated proteins involved in fatty acid degradation and oxidative metabolism positively correlated with both relative PFO and Fat_max_. Multiple regression analysis demonstrated that variability in relative PFO and Fat_max_ were explained by skeletal muscle mitochondrial abundance rather than ${\dot{V}}_{O_{2}\mathrm{peak}}$. Absolute PFO was positively correlated with absolute ${\dot{V}}_{O_{2}\mathrm{peak}}$, which together with mitochondria abundance and muscle glycogen, explained 56% of the variability. Our non‐targeted approach to examine the skeletal muscle proteome highlight the close association between skeletal muscle mitochondrial abundance and both PFO and Fat_max_.

The present cohort demonstrated substantial inter‐individual variability in PFO (>3‐fold), despite consisting of a relatively homogeneous sample of young, physically active males with high ${\dot{V}}_{O_{2}\mathrm{peak}}$ values (average 55.6 mL kg^−1^ min^−1^). This allowed us to examine the relationship between PFO and the skeletal muscle proteome. Interestingly, numerous proteins associated with relative PFO (51 proteins), whereas only two proteins associated with absolute PFO, suggesting that skeletal muscle characteristics have a greater influence on relative PFO. Pathway analysis demonstrated that most of the identified proteins were involved in metabolic pathways including the tricarboxylic acid (TCA) cycle and oxidative phosphorylation, as well as mitochondrial transport and organisation. As such, both the summed mitochondrial proteome and CS activity, another well recognised marker of mitochondrial content (Larsen et al., 2012), were positively associated with relative PFO. Several smaller studies have explored these relationships. Two studies show significant associations between PFO and mitochondrial abundance in young males assessed with transmission electron microscopy (Dandanell et al., 2018) and/or CS activity (Dandanell et al., 2018; Maunder et al., 2022). However, this is not a universal finding, with other studies showing no significant associations between PFO and CS activity in males or females (Nordby et al., 2006; Stisen et al., 2006). A strength of the present study is that we report PFO as the average from two separate measurements, which has been shown to be a more reliable assessment of PFO given the day‐to‐day variability (Chrzanowski‐Smith et al., 2020). Furthermore, the present study includes a larger sample size (*n* = 34) of a relatively homogenous group in terms of sex, age, aerobic capacity and training status. This contrasts previous studies which pooled data (*n* = 15–17) from disparate groups of untrained and endurance trained individuals, which may increase the probability that observed associations are influenced by characteristics related to training status, rather than reflecting intrinsic skeletal muscle determinants of PFO (Dandanell et al., 2018; Nordby et al., 2006; Shaw et al., 2020; Stisen et al., 2006).

Given that ${\dot{V}}_{O_{2}\mathrm{peak}}$ is commonly linked to mitochondrial abundance, it is relevant to consider whether the relationship between PFO and mitochondria abundance simply reflects aerobic capacity. Absolute PFO (g min^−1^) positively correlated with absolute ${\dot{V}}_{O_{2}\mathrm{peak}}$ (Fig. 1*C* and *D*). Interestingly, ${\dot{V}}_{O_{2}\mathrm{peak}}$ and mitochondrial abundance were not significantly associated (*r* = 0.194, *P = *0.271), which is consistent with previous work (Lundby & Jacobs, 2016) and with the central proposition that ${\dot{V}}_{O_{2}\mathrm{peak}}$ is primarily constrained by central limitations rather than skeletal muscle (Lundby et al., 2017; Mortensen et al., 2005). Stepwise multiple regression analysis revealed that both mitochondrial abundance and ${\dot{V}}_{O_{2}\mathrm{peak}}$ independently associated with absolute PFO (Table 1), suggesting that total workload and/or energy expenditure relating to ${\dot{V}}_{O_{2}\max}$, in combination with mitochondrial content, strongly influence absolute PFO.

By contrast, relative PFO was not correlated with absolute nor relative ${\dot{V}}_{O_{2}\mathrm{peak}}$ (Fig. 1*C*). Multiple regression analysis indicated that mitochondrial abundance, but not ${\dot{V}}_{O_{2}\mathrm{peak}}$, independently associated with relative PFO (Table 1). Similarly, the mitochondrial proteome also independently related to Fat_max_ (Table 1), reflecting the substantial overlap between proteins correlating with these variables (Fig. 4*G*). Collectively, these findings highlight the key role of skeletal muscle mitochondrial content in determining substrate utilisation. As the site of β‐oxidation, the TCA cycle and subsequent oxidative phosphorylation, mitochondria are clearly mechanistically relevant for fatty acid oxidation rates during exercise. The potential importance of mitochondria abundance and/or function in PFO has been discussed previously, with suggestions that PFO, in combination with the assessment of lactate threshold, may provide a non‐invasive assessment of mitochondrial content and/or function (San‐Millán & Brooks, 2018). The consistent and independent association between mitochondrial abundance, PFO and Fat_max_ further support this contention.

Mitochondrial functional characteristics have been linked with whole body fat oxidation previously (Sahlin et al., 2007). It was notable that, in the present study, complex specific analysis highlighted that mitochondrial complex V abundance was consistently correlated with both relative PFO and Fat_max_. ATP synthase (complex V) is responsible for the final step in oxidative phosphorylation, utilising the proton gradient to phosphorylate ADP to ATP, and correlates with mitochondrial abundance and mitochondrial respiratory capacity in human muscle (Larsen et al., 2012; Mancilla et al., 2023), as well as functional outcomes including ${\dot{V}}_{O_{2}\max}$ (Emanuelsson et al., 2024) and insulin sensitivity (Kjaergaard et al., 2025). However, it is not immediately clear why complex V is most closely related to PFO which occurs at a low–moderate intensity of exercise (∼40% ${\dot{V}}_{O_{2}\mathrm{peak}}$ in the present study). The modest association between complex V and PFO/Fat_max_ may simply reflect the high abundance of these components detected in the samples and the relationship with total mitochondrial abundance. However, there has recently been significant attention linking complex V function and mitochondrial morphology because it is an important regulator of cristae remodelling. Dimerisation of ATP synthase subunits induces curvature of the inner mitochondrial membrane, contributing to cristae stability and influencing oxidative phosphorylation and cellular metabolism (Cogliati et al., 2016; Yu et al., 2025). However, associations between relative PFO and other cristae remodelling proteins, such as those in the MICOS complex (APOOL, MICOS13, APOO, IMMT and CHCHD3) and OPA1, were modest (*r* < 0.5) and not significant after correction for multiple comparisons (see Supporting information, Table S2). Further work is required to examine the direct influence of cristae remodelling on substrate utilisation in human skeletal muscle.

Given that type I muscle fibres are characterised by high oxidative capacity, enhanced lipid storage and abundance of proteins mediating lipid oxidation (Shaw et al., 2020), muscle fibre type composition has been proposed as a determinant of PFO. Indeed, in previous studies, we and others have shown an association between the proportion of type I fibres and PFO (Dandanell et al., 2018; Shaw et al., 2020). However, in these studies, this relationship was only present when groups of endurance trained and untrained individuals were combined. In the present study, there was no association between abundance of any myosin heavy chain isoforms and either absolute or relative PFO (see Supporting information, Table S2). These data agree with other studies that have reported similar results when examining muscle fibre type composition in females (Stisen et al., 2006), as well as with further studies when looking only at well trained, physically active males (Dandanell et al., 2018; Shaw et al., 2020). Therefore, muscle fibre type does not appear to be a major determinant of PFO, at least when examined in a group of well‐trained individuals.

We also explored relationships between circulating and intramuscular substrate availability and PFO. There was no association between circulating free fatty acids (FFA) and either absolute or relative PFO or Fat_max_. This is somewhat surprising given the rise in PFO over the course of repeated exercise tests is closely associated with elevations in plasma FFA (Frandsen et al., 2021). However, in this context, it may be more relevant to examine plasma FFA concentrations during exercise. Furthermore, neither IMTG nor muscle glycogen content were associated with PFO or Fat_max_. However, when combined with other key variables in the multiple regression analysis, muscle glycogen was independently and negatively associated with absolute and relative PFO. The link between muscle glycogen content and fat oxidation rates is well described (Gollnick et al., 1981; Wojtaszewski et al., 2003) and was further highlighted recently in patients with McArdle's disease, who are unable to utilise muscle glycogen and exhibit very high rates of fat oxidation that occur at a high exercise intensity (Rodriguez‐Lopez et al., 2023).

The close association between PFO and markers of mitochondria abundance was consistent whether we examined CS activity or the summed mitochondrial proteome. CS activity has been shown to be one of the more reliable markers of mitochondrial content (Larsen et al., 2012) and we observed a close association between CS activity and mitochondrial proteome abundance (*r* = 0.725), suggesting that the skeletal muscle mitochondrial content can be reliably estimated from proteomic datasets. The proteomic approach used in the present study allowed us to examine the relationship between PFO and ∼2000 proteins identified in these skeletal muscle samples. Alternate proteomics approaches using data independent acquisition and sample fractionation might have yielded greater proteome coverage (Wong et al., 2025). Related to this, certain key proteins regulating the transport, storage and breakdown of lipids that have been associated with PFO previously (Chrzanowski‐Smith, Edinburgh, Smith et al., 2021; Maunder et al., 2022; Shaw et al., 2020) were not detected in this data set. Consequently, we are unable to determine the relative importance of these (or other low abundant proteins) to PFO. Furthermore, we have not explored the potential role of post‐translational modifications that may alter mitochondrial function in mediating fat oxidation capacity. Although the present study has a relatively large sample size in the examination of skeletal muscle contribution to PFO (*n* = 34), it was not sufficient to explore the contribution of muscle characteristics alongside the broad range of physiological, dietary and physical activity variables previously linked with PFO. In addition, we only examined males, which is a significant limitation to the current dataset. There are well described sex differences in substrate utilisation with females having a higher contribution of fat during exercise (Tarnopolsky et al., 1990) and higher reported relative PFO (Randell et al., 2017; Venables et al., 2005). There are also potential differences in plasma and muscle substrate utilisation (Devries, 2016), which, collectively, could influence the physiological determinants of PFO and Fat_max_. Unfortunately completing these studies in males and females exceeded the available resources meaning further studies exploring PFO and skeletal muscle characteristics in females is essential.

In the present study, we used a non‐targeted mass‐spectrometry based proteomics approach to examine the relationship between PFO and the skeletal muscle proteome. We demonstrate that, in a young and moderate‐well trained male cohort, the abundance of the mitochondrial proteome is independently associated with both relative and absolute PFO, as well as Fat_max_. Importantly, ${\dot{V}}_{O_{2}\mathrm{peak}}$ was not independently associated with relative PFO or Fat_max_, indicating that skeletal muscle mitochondrial abundance is particularly important for determining substrate selection during exercise. These findings add to the existing body of work by demonstrating that muscle oxidative capacity contributes to the interindividual variability in PFO.

## Additional information

## Competing interests

The authors declare that they have no competing interests.

## Author contributions

E.K.T., S.M., G.A.K., S.G.V., E.A.P., M.C.O., M.H., A.I., K.F.H., S.L., C.S.S. and J.W.H. were responsible for study conception and design. E.K.T., S.M., S.G.V., E.A.P., M.C.O., M.H. and A.I. were responsible for data collection. E.K.T., S.M., G.A.K., S.G.V. and C.S.S. were responsible for data analysis. E.K.T., C.S.S. and J.W.H. were responsible for drafting the manuscript. S.M., G.A.K., S.G.V., E.A.P., M.C.O., M.H., A.I., K.F.H. and S.L. were responsible for critically reviewing the manuscript for intellectual content. E.K.T., S.M., G.A.K., S.G.V., E.A.P., M.C.O., M.H., A.I., K.F.H., S.L., C.S.S. and J.W.H. were responsible for approving the final version of the manuscript submitted for publication. E.K.T., S.M., G.A.K., S.G.V., E.A.P., M.C.O., M.H., A.I., K.F.H., S.L., C.S.S. and J.W.H. agree to be accountable for all aspects of the work. All human trials were performed at the University of Copenhagen. Sample analysis was performed at the University of Copenhagen and Deakin University.

## Funding

Funding was received from Aase and Ejnar Danielsens Foundation and the Center of Healthy Aging.

## Supporting information




Peer Review History

Supporting Information

## Data Availability

The proteomics data have been deposited to the ProteomeXchange Consortium via the PRIDE partner repository (dataset identifier: PXD064359 and https://doi.org/10.6019/PXD064359). The physiological data are available upon request.
