# Supplementary material for: Skeletal muscle proteomics links mitochondrial abundance with peak fat oxidation in physically active young males
Source: J Physiol. 2026 May 28;604(14):5936–51. doi: 10.1113/JP290966 (PMC13370683; doi:10.1113/JP290966)
Supplement: Supplementary file 1 — Peer Review History [file TJP-604-5936-s002.pdf]

The Journal of Physiology JP

Skeletal Muscle Proteomics Links Mitochondrial Abundance with Peak Fat Oxidation in Physically Active Young Males

Eloise Kate Tarry, Shaun Mason, Ghazanfar Abbas Khan, Sofie Greve Vestergaard, Emilie Ahmt Petersen, Mike Cadovius Olsen, Maria Hansen, Arthur Ingersen, Kirsten F Howlett, Steen Larsen, Christopher Shaw, and Jørn Wulff Helge
DOI: 10.1113/JP290966

Corresponding author(s): Christopher Shaw (chris.shaw@deakin.edu.au)

The following individual(s) involved in review of this submission have agreed to reveal their identity: Ed Maunder (Referee #1); Malene Lindholm (Referee #2)

Review Timeline:

Submission Date:	20-Jan-2026
Editorial Decision:	17-Feb-2026
Revision Received:	07-Apr-2026
Accepted:	06-May-2026

Senior Editor: Paul Greenhaff

Reviewing Editor: Mark Viggars

Transaction Report:

(Note: With the exception of the correction of typographical or spelling errors that could be a source of ambiguity, letters and reports are not edited. Depending on transfer agreements, referee reports obtained elsewhere may or may not be included in this compilation. Referee reports are anonymous unless the Referee chooses to sign their reports.)

Re: JP-RP-2026-290966 "**Skeletal Muscle Proteomics Links Mitochondrial Abundance with Peak Fat Oxidation in Physically Active Young Males**" by Eloise Kate Tarry, Shaun Mason, Ghazanfar Abbas Khan, Sofie Greve Vestergaard, Emilie Ahmt Petersen, Mike Cadovius Olsen, Maria Hansen, Arthur Ingersen, Kirsten F Howlett, Steen Larsen, Christopher Shaw, and Jørn Wulff Helge

Dear Dr Shaw,

Thank you for submitting your manuscript to The Journal of Physiology. It has been assessed by a Reviewing Editor and by 2 expert referees and we are pleased to tell you that it is potentially acceptable for publication following satisfactory major revision.

Please advise your co-authors of this decision as soon as possible.

The referee reports are copied at the end of this email.

Please address all the points raised and incorporate all requested revisions or explain in your Response to Referees why a change has not been made. We hope you will find the comments helpful and that you will be able to return your revised manuscript within 2 months. If your article is NOT for a Special Issue, you may have 9 months to revise. If you require an extension, please contact journal staff: jp@physoc.org. Please note that this letter does not constitute a guarantee for acceptance of your revised manuscript.

Your revised manuscript should be submitted online using the link in your Author Tasks: Link Not Available. This link is accessible via your account as Corresponding Author; it is not available to your co-authors. If this presents a problem, please contact journal staff (jp@physoc.org). Image files from the previous version are retained on the system. Please ensure you replace or remove any files that are being revised.

If you do not wish to submit a revised version of your manuscript, you must inform our journal staff (jp@physoc.org) or reply to this email to request withdrawal. Please note that a manuscript must be formally withdrawn from the peer review process at one journal before it may be submitted to another journal.

TRANSPARENT PEER REVIEW POLICY: To improve the transparency of its peer review process, The Journal of Physiology publishes online as supporting information the peer review history of all articles accepted for publication. Readers will have access to decision letters, including Editors' comments and referee reports, for each version of the manuscript, as well as any author responses to peer review comments. Referees can decide whether or not they wish to be named on the peer review history document.

ABSTRACT FIGURES: Authors are expected to use The Journal's premium BioRender account to create/redraw their Abstract Figures. Information on how to access this account is here: https://physoc.onlinelibrary.wiley.com/journal/14697793/biorender-access.

This will enable Authors to create and download high-resolution figures. If authors have used the free BioRender service, they can use the instructions provided in the link above to download a high-resolution version suitable for publication.

The link provided should only be used for the purposes of this submission. Authors will be charged for figures created on this account if they are not related to this manuscript submission.

LANGUAGE EDITING AND SUPPORT FOR PUBLICATION: If you would like help with English language editing, or other article preparation support, Wiley Editing Services offers expert help, including English Language Editing, as well as translation, manuscript formatting, and figure formatting at www.wileyauthors.com/eeo/preparation. You can also find resources for Preparing Your Article for general guidance about writing and preparing your manuscript at www.wileyauthors.com/eeo/prepresources.

RIGOUR AND REPRODUCIBILITY: We recommend authors consult The Journal's Rigour and Reproducibility requirements to ensure that their manuscript is suitable for publication: https://physoc.onlinelibrary.wiley.com/pb-assets/hub-assets/physoc/documents/TJP-Rigour-and-Reproducibility-Requirements.pdf

REVISION CHECKLIST:

Check that your Methods section conforms to journal policy: https://jp.msubmit.net/cgi-bin/main.plex?form_type=display_requirements#methods.

Check that data presented conforms to the statistics policy: https://jp.msubmit.net/cgi-bin/main.plex?form_type=display_requirements#statistics.

Upload a full Response to Referees file. To create your 'Response to Referees': copy all the reports, including any comments from the Senior and Reviewing Editors, into a Microsoft Word, or similar, file and respond to each point, using

font or background colour to distinguish comments and responses and upload as the required file type.

Please upload two versions of your manuscript text: one with all relevant changes highlighted and one clean version with no changes tracked. The manuscript file should include all tables and figure legends, but each figure/graph should be uploaded as separate, high-resolution files.

You may also upload:

- 'Potential Cover Art' for consideration as the issue's cover image
- Appropriate Supporting Information (Video, audio or data set: see https://jp.msubmit.net/cgi-bin/main.plex?form_type=display_requirements#supp).

We look forward to receiving your revised submission.

If you have any queries, please reply to this email and we will be pleased to advise.

Yours sincerely,

Paul Greenhaff
Senior Editor
The Journal of Physiology

REQUIRED ITEMS

- Author photo and profile. First or joint first authors are asked to provide a short biography (no more than 100 words for one author or 150 words in total for joint first authors) and a portrait photograph. These should be uploaded and clearly labelled together in a Word document with the revised version of the manuscript. See Information for Authors for further details.

- You must start the Methods section with a paragraph headed Ethical Approval. If experiments were conducted on humans, confirmation that informed consent was obtained, preferably in writing, that the studies conformed to the standards set by the latest revision of the Declaration of Helsinki and that the procedures were approved by a properly constituted ethics committee, which should be named, must be included in the article file. If the research study was registered (clause 35 of the Declaration of Helsinki), the registration database should be indicated, otherwise the lack of registration should be noted as an exception (e.g. The study conformed to the standards set by the Declaration of Helsinki, except for registration in a database). For further information see: https://physoc.onlinelibrary.wiley.com/hub/human-experiments.

- The reference list must be in alphabetical order, rather than numbered, to comply with our Journal format.

- Your manuscript must include a complete Additional Information section, including competing interests; funding; author contributions and acknowledgements.

- Please upload separate high-quality figure files via the submission form.

- Please include an Abstract Figure file and an Abstract Figure legend. An appropriate figure legend, which should not exceed 150 words in length, should be included in the main manuscript file. The Abstract Figure is a piece of artwork designed to give readers an immediate understanding of the research and should summarise the main conclusions. If possible, the image should be easily 'readable' from left to right or top to bottom. It should show the physiological relevance of the manuscript so readers can assess the importance and content of its findings. Abstract Figures should not merely recapitulate other figures in the manuscript. Please try to keep the diagram as simple as possible and without superfluous information that may distract from the main conclusion(s). Abstract Figures must be provided by authors no later than the revised manuscript stage and should be uploaded as a separate file during online submission labelled as File Type 'Abstract Figure'. Please also ensure that you include the figure legend in the main article file. All Abstract Figures should be created using BioRender. Authors should use The Journal's premium BioRender account to export high-resolution images. Details on how to use and access the premium account are included as part of this email.

EDITOR COMMENTS

Reviewing Editor: Comments to the Author (Required):
Thankyou for your submission to The Journal of Physiology. Your manuscript has been reviewed by two experts in the field who both commended the large body of work completed in this study.

While there is a substantive prior literature supporting a link between mitochondrial content/function and fat oxidation, this study clearly adds novelty using untargeted skeletal muscle proteomics. A number of concerns were raised that limit the enthusiasm for this work currently in The Journal of Physiology.

-Despite interindividual variation in PFO, the findings are limited to young, healthy males and cannot be extrapolated to females or different disease states. Furthermore, this is a fasted exercise bout, with no information on habitual diet composition/timing or recent exercise history.

-Having two visits is a strength of the study, but the biopsy data is from Visit 2. There is lack of discussion between visit 1 and visit 2 PFO variation which may weaken/strengthen relationships. Given day-to-day variability in mitochondrial function, is 4-weeks too long to study relationships between PFO and the proteome?

-Whole-muscle, untargeted proteomics yields relative abundances and biases high-abundance proteins. There is lack of information on the potential importance of lower abundance regulators and post-translational modifications (PTMs) that are critical for mitochondrial function, PMID: 38701776.

-Whole-body gas exchange gives PFO but does not partition muscle vs. non-muscle contributions or mitochondrial substrate flux at the cellular level. Mitochondrial function assays to measure oxidative flux, coupling efficiency, substrate preference, or maximal respiratory capacity) would be required to improve causality.

Senior Editor:

Comments to the Author:
Thank you for the manuscript submission to The Journal of Physiology which has been considered by a reviewing editor and two reviewers. All were in agreement that there were several positive aspects about the study and that the topic is of interest to the readership of J Physiology, but Referee 2 and the Reviewing Editor both raised several concerns regarding the novelty of the work and the lack of robust evidence of causality. The authors are invited to address these concerns, which may involve additional experimental work. WE look forward to receiving the revised version of the manuscript along with an author response document.

From an ethics perspective, please ensure a statement is made in the Methods section that the study conformed to the standards set by the latest version of the Declaration of Helsinki or the version that was in place at the time of the experiments). If the research study was registered (clause 35 of the Declaration of Helsinki) the registration database should be indicated, else the lack of registration should be noted as an exception (e.g. The study conformed to the standards set by the Declaration of Helsinki, except for registration in a database.) Please also ensure exact P values (not < or >) are provided throughout.

REFEREE COMMENTS

Referee #1:

Thanks for the opportunity to review this excellent and well-written manuscript. Please find my comments in the attached.

Referee #2:

This is an interesting study that aims to investigate the skeletal muscle proteins that contribute to peak absolute and relative fat oxidation, a physiological variable of great interest for health and performance. The strengths of the study includes repeated measurements of peak FAO, a reasonably sized cohort (N=34) and the use of untargeted proteomics to assess the skeletal muscle proteome.

Comments/concerns

While there was some variability in the PFO and FatMax in this homogeneous population, when the purpose was to identify muscle proteins that can explain some of the variability in these variables, wouldn't it make more sense to include people with more variable PFO?

Why were only male subjects included?

For the phenotypic correlations, was the reported p-value adjusted for multiple testing? Considering the number of correlation analyses performed, that should be done.

Since absolute and relative PFO correlates with absolute and relative peak VO2, do the correlated proteins also correlate with peak VO2 and are these training-induced proteins that reflect better fitness rather than fatty acid oxidation per se? Most of them are mitochondrial and perhaps reflect mitochondrial abundance/volume?

What was the reason for doing the stepwise multiple linear regression analysis? All of these variables share considerable collinearity and the analysis cannot distinguish between direct and indirect effects. It could be that the mitochondrial proteome change with training is what is explaining both the VO2peak and the PFO.

The study claims to provide a more reliable measure of PFO due to two measures as there is high day-to-day variability - if the proposed proteins are important for PFO, how come two matching measurements of proteomics was not performed? What is the day to day variability in the proteome? If the proposed correlated proteins were different in the same direction as the PFO at different time points, this would have provided much stronger evidence for a causal link to PFO.

When was the mass-spec analysis performed? 2000 proteins detected are quite few.

END OF COMMENTS

Tarry et al.

Skeletal muscle proteomics links mitochondrial abundance with peak fat oxidation in physically active young males

Congratulations on a nice study, and thank you for the opportunity to review it. The manuscript is very well-written, with clear rationale, a clear research question, appropriate and rigorous methods, and clear results. It is challenging to present results clearly from an omics study, so the authors are to be congratulated for this.

My comments are relatively minor—see specific comments below. It would be particularly useful to have access to the proteomic data in a supplementary file, and for the authors to offer some explanation for why proteins involved in lipid handling were not detected.

Specific comments

Title and abstract

- The abstract is good, but would be more helpful if key data were included (e.g. correlation values for PFO and VO_2peak, multiple regression outputs, etc. I appreciate that you can't cite every r value in the abstract, but a couple that speak to the strength of the relationships that are primary components of the conclusion would be helpful.

Introduction

- The study is well-rationalised in the introduction. The main purpose of the study is to build on previous work by taking a more comprehensive approach to assessing the skeletal muscle protein characteristics that are associated with peak fat oxidation during exercise, using untargeted proteomics.
- One of the critiques of previous work made in the introduction was the relatively heterogenous groups. Can you please explain why this is a weakness in an associational study? I'm not debating that it is critical to interpretation, but I think if this point is to be made, it should be made more clearly.

Methods

- L142: "3rd" should be "third".
- There should be a space between values and W, i.e. 95 W rather than 95W.
- L151: Can you cite work to support this second-degree polynomial approach?
- L161-165: Minimal blood data is not presented in this manuscript, but 50 mL was collected. I presume this was collected as part of another study? If so, please state.
- L184" "mins" should be "min".
- L198: "@" should be "at".
- The methods are extremely well-described, well done.

Results

- L276-287: Some of these data are already presented in the methods.

- Was there any systematic variation in PFO etc between visit 1 and 2? I note visit 2 was preceded by a muscle biopsy and blood sampling. I think it would be good to note, even if there is no difference (as is my guess)—this will help future researchers justify that a pre-assessment biopsy does not influence subsequent incremental test outcomes.
- L314: "2" should be "two".
- Again, the results are presented clearly, well done.
- Is it possible to make the individual uncorrected and corrected correlations available for all proteins in a supplementary file? One of the challenges with these types of studies is communicating the (many) outcomes, and this seems to me an appropriate means of doing so. I have been unable to access the data using the ProteomeXchange Consortium.

Discussion

- L469-471: This sentence doesn't read very well.
- L485-488: I don't necessarily disagree, but your point that a more homogenous group is favourable for answering this research question requires greater elucidation.
- L500: Could this be explained by data demonstrating that VO2peak is not likely to be limited by mitochondrial protein abundance—i.e. that VO2peak is limited by oxygen delivery? This suggests that they are independent. See: https://pubmed.ncbi.nlm.nih.gov/15860533/
- L559-561: Why do you think they weren't detected?

EDITOR COMMENTS

Reviewing Editor: Comments to the Author (Required):
Thankyou for your submission to The Journal of Physiology. Your manuscript has been reviewed by two experts in the field who both commended the large body of work completed in this study.

While there is a substantive prior literature supporting a link between mitochondrial content/function and fat oxidation, this study clearly adds novelty using untargeted skeletal muscle proteomics. A number of concerns were raised that limit the enthusiasm for this work currently in The Journal of Physiology.

Thank you for your constructive feedback. Your comments have helped improve the clarity and overall quality of the manuscript, and we appreciate the time dedicated to reviewing our work. The corresponding revisions made to the manuscript are outlined in detail below and the line and page details refer to the revised manuscript with marked up changes in red.

Despite interindividual variation in PFO, the findings are limited to young, healthy males and cannot be extrapolated to females or different disease states.

We acknowledge that the findings are limited to young, healthy males. However, inclusion of female participants was not feasible in the present study. Given the known sex differences in substrate utilisation, and specifically peak fat oxidation (PFO) relative to fat-free mass, adequately accounting for these differences would have required either a separate, sex-specific study or a substantially larger sample size to ensure appropriate statistical power. To adequately account and control for hormonal variations across the menstrual cycle, sufficient high-quality cycle classification and tracking is warranted (Smith et al., 2025. PMID: 41297531) adding to the study complexity. These options were beyond the scope and available resources of the current investigation. We recognise that exploring these research questions using similar approaches in females is both important and warranted. However, conducting such work requires a dedicated study design specifically powered and structured to address these questions comprehensively. These limitations have been acknowledged and explained in the discussion on page 20 lines 547.

Furthermore, this is a fasted exercise bout, with no information on habitual diet composition/timing or recent exercise history.

An overnight fasted state (10-12 hours) was utilised as this represents the standardised approach for accurately determining peak fat oxidation (PFO) (Achten et al., 2002. PMID: 11782653) and is the most widely utilised methodology (Amaro-Gahete et al., 2019. PMID: 30929281). We acknowledge that this may limit the direct applicability of the findings to exercise performed in a fed state. However, the primary aim of the study was not to examine performance or responses under typical feeding conditions, but rather to explore the relationship of the muscle proteome with PFO, using the most validated measure for its assessment.
We have collected data to estimate energy intake using MADLOG ApS (Kolding, Denmark),

which integrates a comprehensive food database particularly catered towards the Danish market. Habitual physical activity and energy expenditure were monitored for four days using an activity watch (Fitbit Charge 4, San Francisco, CA, USA). Details have now been added to the methods (line 181) and results section (line 293). Furthermore, correlations with energy intake and macronutrient intake have been added to Figure 1C.

Having two visits is a strength of the study, but the biopsy data is from Visit 2. There is lack of discussion between visit 1 and visit 2 PFO variation which may weaken/strengthen relationships. Given day-to-day variability in mitochondrial function, is 4-weeks too long to study relationships between PFO and the proteome?

Thank you for highlighting this. While we endeavoured to minimise the duration between Visit 1 and Visit 2, this was not always possible given the practical constraints inherent to invasive research in humans and the fact that this study was completed during COVID-related restrictions. Importantly, we observed no systematic variation in PFO between Visit 1 and Visit, figure below. We have included the mean PFO and Fatmax from visits 1 and 2 in the latest version of the paper (line 301 and 307).

We have also examined the correlation between mitochondrial proteome abundance and relative PFO in visits 1 and 2. The correlations were similar from the PFO data in visit 1 (r = 0.443) and visit 2 (r = 0.489), illustrating that the gap between visits had little effect on the relationship between PFO and mitochondrial abundance. Notably the correlations using the average PFO values from the 2 visits substantially strengthened the correlation (r = 0.633, as reported in Figure 3), demonstrating the advantage of repeated measurement of PFO.

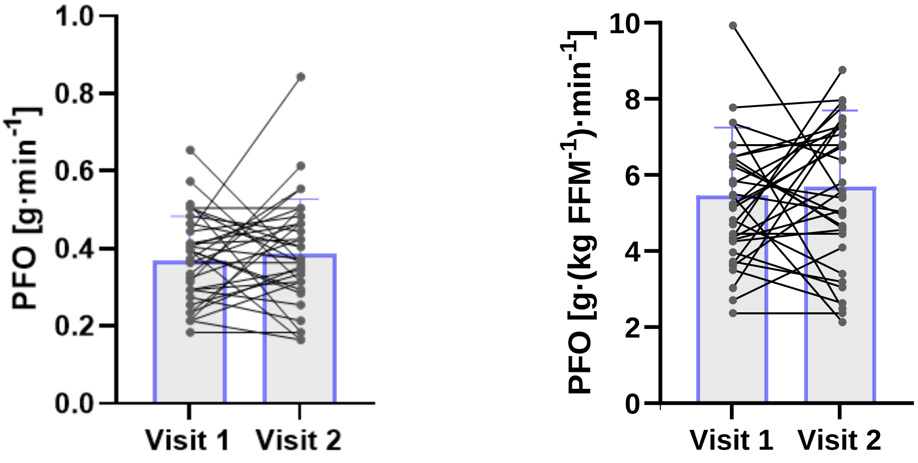

Figure 1. Variation in Peak Fat Oxidation between visits 1 and 2.

Whole muscle, untargeted proteomics yields relative abundances and biases high abundance proteins. There is lack of information on the potential importance of lower abundance

regulators and posttranslational modifications (PTMs) that are critical for mitochondrial function, PMID: 38701776.

It is important to restate that the findings relate only to mitochondrial abundance (assessed by both proteomics and CS activity) rather than mitochondrial function. Indeed, previous studies have shown there is no clear relationship between PFO and intrinsic mitochondrial function (Dandanell et al. 2018. PMID: 30218613)

Nevertheless, we acknowledge that we cannot rule out the importance of lower abundance proteins or post-translational modifications (e.g. acetylation) in mediating the relationship between PFO and skeletal muscle but this was beyond the scope of the current study. This limitation has been included in the discussion in line 540.

Whole body gas exchange gives PFO but does not partition muscle vs. Non muscle contributions or mitochondrial substrate flux at the cellular level. Mitochondrial function assays to measure oxidative flux, coupling efficiency, substrate preference, or maximal respiratory capacity) would be required to improve causality.

We appreciate this insightful suggestion. Our primary objective was to specifically investigate how whole-body PFO relates to the proteome, to extend the current body of work on PFO at the whole-body level. Assessment of the contribution of muscle and plasma-derived sources requires several assumptions, invasive measures and could not be measured during the short incremental PFO test. This is an important question that was beyond the scope and resources available for this project.

Senior Editor:

Comments to the Author:
Thank you for the manuscript submission to The Journal of Physiology which has been considered by a reviewing editor and two reviewers. All were in agreement that there were several positive aspects about the study and that the topic is of interest to the readership of J Physiology, but Referee 2 and the Reviewing Editor both raised several concerns regarding the novelty of the work and the lack of robust evidence of causality. The authors are invited to address these concerns, which may involve additional experimental work. WE look forward to receiving the revised version of the manuscript along with an author response document.

Thank you for the positive comments relating to the manuscript and the opportunity to address the reviewers comments/concerns. We have responded to the individual reviewer comments below.

From an ethics perspective, please ensure a statement is made in the Methods section that the study conformed to the standards set by the latest version of the Declaration of Helsinki or the version that was in place at the time of the experiments). If the research study was registered (clause 35 of the Declaration of Helsinki) the registration database should be indicated, else the lack of registration should be noted as an exception (e.g. The study conformed to the standards set by the Declaration of Helsinki, except for registration in a database.)

Thanks for bringing this to our attention. Please find the ethics statement amended on line 128 within the methods section to include the study registration and alignment with the Declaration of Helsinki (2013).

Please also ensure exact P values (not < or >) are provided throughout.

P values have been updated to exact values where appropriate (when $P > 0.0001$). For multi-regression analysis performed in SPSS, exact p values are reported when $P > 0.001$ as per the output in SPSS. Figures have also been updated to include exact P values.

REFEREE COMMENTS

Referee #1:

Thanks for the opportunity to review this excellent and well-written manuscript. Please find my comments in the attached.

Congratulations on a nice study, and thank you for the opportunity to review it. The manuscript is very well-written, with clear rationale, a clear research question, appropriate and rigorous methods, and clear results. It is challenging to present results clearly from an omics study, so the authors are to be congratulated for this. My comments are relatively minor—see specific comments below.

It would be particularly useful to have access to the proteomic data in a supplementary file, and for the authors to offer some explanation for why proteins involved in lipid handling were not detected.

We did include the full proteomic dataset as a supplementary file in the submission, this reports all proteins detected and information on the R value as well as raw and corrected P values.

There is always variability in the number and specific proteins detected in tissues samples using untargeted proteomics, and the presence of higher abundant proteins in skeletal muscle (e.g. contractile and structural proteins) may prevent detection of lower abundant proteins. As mentioned in our response to reviewer 2 below, and in the text (line 542), alternate sample preparation and data acquisition methods may have yielded a higher protein coverage (Wong et al. PMID: 40604168). We should clarify that many lipid handling proteins were detected and pathway analysis demonstrated lipid metabolic processes were related to Fatmax in particular (Figure 4). However, some common proteins, such as CD36/FAT and other fatty acid transport proteins were not detected which we consider to be a limitation as mentioned in the discussion.

Specific comments

Title and abstract

• The abstract is good, but would be more helpful if key data were included (e.g. correlation values for PFO and VO2peak, multiple regression outputs, etc. I appreciate that you can’t cite every r value in the abstract, but a couple that speak to the strength of the relationships that are primary components of the conclusion would be helpful.

We agree that these additions would strengthen the abstract. We have added key statistical outcomes that relate to the strength of key associations and have made some minor edits to comply with the word count.

Introduction

• The study is well-rationalised in the introduction. The main purpose of the study is to build on previous work by taking a more comprehensive approach to assessing the skeletal muscle

protein characteristics that are associated with peak fat oxidation during exercise, using untargeted proteomics.

• One of the critiques of previous work made in the introduction was the relatively heterogenous groups. Can you please explain why this is a weakness in an associational study? I'm not debating that it is critical to interpretation, but I think if this point is to be made, it should be made more clearly.

A limitation of prior work has been inclusion of small groups (n=7-9) of untrained and endurance trained individuals which results in a number of skeletal muscle variables correlating with PFO across all individuals. These results may simply reflect the group differences resulting from the divergent training status. It is notable that many of these associations are not present when a single group is analysed. Therefore, we wanted to examine a group with similar characteristics and a narrower range of VO2max. This approach has shown that mitochondrial abundance is more strongly related to relative PFO and Fatmax, than other training-related variables, including VO2max.

On reflection, the term heterogenous may be misleading and is could be interpreted as a broad spectrum of characteristics, when the limitation of previous work is examining correlations across two or more disparate groups. To clarify this, we have amended and extended the rationale in the introduction (lines 105) and in the discussion of the studies strengths (lines 435).

Methods

• L152: "3rd" should be "third".

This has been amended

• There should be a space between values and W, i.e. 95 W rather than 95W.

This has been amended within the methods section (physiological phenotyping, Visit 1 paragraph).

• L151: Can you cite work to support this second-degree polynomial approach?

Citations have been provided in line 167. Previous studies have used both 2nd and 3rd order polynomial approaches. Whilst third order fittings allow more flexibility, they are more susceptible to overfitting leading to inflated estimates of PFO (Chrzanowski-Smith et al., 2020, PMID: 32488584). Similar levels of agreement have been reported between analyses methods (Chrzanowski-Smith et al., 2020 PMID: 32488584, Croci et al., 2014 PMID: 24886715). In this instance, we have used the more conservative approach. To date, second-order curve fitting provides the most widely used analyses approach (Amaro-Gahete et al., 2019, PMID: 30929281).

• L161-165: Minimal blood data is not presented in this manuscript, but 50 mL was collected. I presume this was collected as part of another study? If so, please state.

This study uses a subset of samples from a larger study that is published. This has been added to the manuscript (line 137).

• L184” “mins” should be “min”.

This has been amended and adjusted in other instances throughout the methods section.

• L198: “@” should be “at”. – amended.

• The methods are extremely well-described, well done.

Results

• L276-287: Some of these data are already presented in the methods.

This has been amended and removed from the methods.

• Was there any systematic variation in PFO etc between visit 1 and 2? I note visit 2 was preceded by a muscle biopsy and blood sampling. I think it would be good to note, even if there is no difference (as is my guess)—this will help future researchers justify that a pre-assessment biopsy does not influence subsequent incremental test outcomes.

See response in line 53 of this response document – this has been added to the manuscript (line 301 and 307). Thank you for the suggestion.

• L314: “2” should be “two”.

This has been amended.

• Again, the results are presented clearly, well done.

• Is it possible to make the individual uncorrected and corrected correlations available for all proteins in a supplementary file? One of the challenges with these types of studies is communicating the (many) outcomes, and this seems to me an appropriate means of doing so. I have been unable to access the data using the ProteomeXchange Consortium.

See response in line 135 of this document. The data has been included a supplementary data and we have checked the data on Proteome Xchange and this is now available at the included link (line 252).

Discussion

• L469-471: This sentence doesn’t read very well.

This has been amended – now line 413.

• L485-488: I don’t necessarily disagree, but your point that a more homogenous group is favourable for answering this research question requires greater elucidation

Related to your previous point above, we have amended the rationale (lines 105) and discussion (lines 435) to clarify this point.

• L500: Could this be explained by data demonstrating that VO2peak is not likely to be limited by mitochondrial protein abundance—i.e. that VO2peak is limited by oxygen delivery? This suggests that they are independent. See: https://pubmed.ncbi.nlm.nih.gov/15860533/

Yes, we fully agree with this point. Along with changes to clarify other points from the review, we have refined the discussion and included this point in lines 459 onwards.

• L559-561: Why do you think they weren't detected?

See response to your comment on line 135 of this document.

Referee #2:

This is an interesting study that aims to investigate the skeletal muscle proteins that contribute to peak absolute and relative fat oxidation, a physiological variable of great interest for health and performance. The strengths of the study includes repeated measurements of peak FAO, a reasonably sized cohort (N=34) and the use of untargeted proteomics to assess the skeletal muscle proteome.

Comments/concerns

While there was some variability in the PFO and FatMax in this homogeneous population, when the purpose was to identify muscle proteins that can explain some of the variability in these variables, wouldn't it make more sense to include people with more variable PFO?

A limitation of previous work examining muscle characteristics is that associations are present when examining relatively small and disparate groups of both untrained and endurance trained individuals. In these studies, numerous muscle characteristics that respond to regular exercise training (fibre type, capillary density, oxidative enzymes etc) associate with PFO, which may simply reflect endurance training adaptations rather than meaningful associations. To illustrate this point further, these relationships are often attenuated or absent when analysed within a single more homogenous group (e.g. Shaw et al. 2020, PMID: 31917629, Dandanell et al. PMID: 30218613, Nordby et al. PMID: 16643200) - albeit with a small sample size. We consider the relative homogeneity of our sample to be a key strength of the study, reducing the impact of potential confounding factors (VO2peak, body composition, age, sex, physical activity) which are known to influence PFO.

It is also worth noting that despite this being a somewhat homogenous group of individuals, there was a 3-4-fold range in absolute PFO/relative PFO, underscoring the substantial interindividual variability in PFO within a homogenous group. We have amended the manuscript to strengthen the rationale (line 105) and is highlighted in the discussion (line 435).

Why were only male subjects included?

Please see the response to the editor in line 18 of this response document.

For the phenotypic correlations, was the reported p-value adjusted for multiple testing? Considering the number of correlation analyses performed, that should be done.

Thank you for this suggestion. We have amended Figure 1C and related text in the results section to include adjustment for multiple comparisons using the Benjamini–Hochberg false discovery rate procedure. This information has also been added to the methods section (line 260) and results have been amended to reflect the data from this analysis (Figure 1C).

Since absolute and relative PFO correlates with absolute and relative peak VO2, do the correlated proteins also correlate with peak VO2 and are these training-induced proteins that reflect better fitness rather than fatty acid oxidation per se?

We consider this to be an important point. We agree in terms of absolute PFO, with this outcome most strongly correlating with absolute VO2max, so higher aerobic capacity and related power output explains some of the variability in absolute PFO even in this homogenous group. Interestingly, mitochondrial abundance was not associated with VO2max (L/min) (r = 0.194, p = 0.271). This is aligned with previous work showing similar lack of associations in well trained individuals (PMID: 26440213) and with long standing hypotheses that VO2max is primarily limited by central mechanisms rather than skeletal muscle factors, including mitochondrial volume (PMID: 27888580). This has been clarified in the discussion in 467.

Furthermore, the correlation between both relative PFO and Fatmax with VO2max (absolute or relative) was weak and not significant (Figure 1C) and the multiple regression analysis (which including VO2max as an independent variable) add strength to our conclusion that mitochondrial abundance is independently related to relative PFO and Fatmax.

Most of them are mitochondrial and perhaps reflect mitochondrial abundance/volume?

Yes, we conclude that the correlation between relative PFO/Fatmax and multiple mitochondrial proteins reflect the relationship with mitochondrial abundance, which is confirmed with the relationship with CS activity (Figure 3I). Collectively, our data show that relative PFO and Fatmax are more strongly related to muscle mitochondrial abundance than VO2max.

What was the reason for doing the stepwise multiple linear regression analysis? All of these variables share considerable collinearity and the analysis cannot distinguish between direct and indirect effects. It could be that the mitochondrial proteome change with training is what is explaining both the VO2peak and the PFO.

Multiple regression analysis was performed in attempt to ascertain the independent relationships between key muscle characteristics and VO2max with capacity for fat oxidation. Collinearity was checked prior to this analysis. Correlations between included variables were, at most, modest (<0.4) and collinearity diagnostics indicated no problematic multicollinearity (all Variance Inflation Factor < 1.5, tolerance > 0.8). This is mentioned in the methods in line 282.

This aligns with the previous comment and response (line 272 in this response document), where mitochondrial abundance did not significantly correlate with VO2max.

The study claims to provide a more reliable measure of PFO due to two measures as there is high day-to-day variability - if the proposed proteins are important for PFO, how come two matching measurements of proteomics was not performed? What is the day to day variability in the proteome? If the proposed correlated proteins were different in the same direction as the PFO at different time points, this would have provided much stronger evidence for a causal link to PFO.

The day-to-day variability in PFO is likely due to a number of nutritional and physiological factors that have been previously shown to contribute to PFO in the published research

literature. Collecting muscle biopsies on two separate occasions for proteome analysis was not performed in this study and as such we are not able to specifically comment on the day-to day variability on the skeletal muscle proteome and whether this would correlate in the same direction as the PFO. However, we would not have expected the proteome to vary substantially across this period given that subjects were active, consumed a normal balanced diet, and that both diet and exercise were well controlled prior to PFO tests and muscle biopsy collection. Furthermore, as explained above (line 53 of this document) there was no systematic variation in PFO between visits, and the proteomic outcomes (mitochondrial abundance) from the single biopsy sample showed a similar relationship with PFO across visits 1 and 3. Furthermore, if multiple biopsies were obtained for separate proteomics analysis we could also not rule variability in the proteome as a result of technical differences in measurements or different muscle biopsy sampling sites.

When was the mass-spec analysis performed? 2000 proteins detected are quite few.

The mass spec analysis was performed in 2024. We are aware that the number of proteins detected is smaller than some recent publications, but the number of proteins detected is in consistent with the approach taken (Wong et al. PMID: 40604168). We acknowledge that alternate proteomics approaches such as data-independent acquisition and fractionation might have yielded greater proteome coverage. This is explained and acknowledged in the discussion (line 536).

1st Revision - Editorial Decision 06-May-2026

Dear Dr Shaw,

Re: JP-RP-2026-290966R1 "**Skeletal Muscle Proteomics Links Mitochondrial Abundance with Peak Fat Oxidation in Physically Active Young Males**" by Eloise Kate Tarry, Shaun Mason, Ghazanfar Abbas Khan, Sofie Greve Vestergaard, Emilie Ahmt Petersen, Mike Cadovius Olsen, Maria Hansen, Arthur Ingersen, Kirsten F Howlett, Steen Larsen, Christopher Shaw, and Jørn Wulff Helge

We are pleased to tell you that your paper has been accepted for publication in The Journal of Physiology.
- Your manuscript must include a complete Additional Information section, including author contributions.

The last Word (or similar) version of the manuscript provided will be used by the Production Editor to prepare your proof. When this is ready you will receive an email containing a link to Wiley's Online Proofing System. The proof should be thoroughly checked and corrected as promptly as possible.

Authors should note that it is too late at this point to offer corrections prior to proofreading. Major corrections at proof stage, such as changes to figures, will be referred to the Editors for approval before they can be incorporated. Only minor changes, such as to style and consistency, should be made at proof stage. Changes that need to be made after proof stage will usually require a formal correction notice.

All queries at proof stage should be sent to: TJP@wiley.com.

Following copyediting and typesetting the accepted version will be published online.

Yours sincerely,

Paul Greenhaff
Senior Editor
The Journal of Physiology

IMPORTANT POINTS TO NOTE FOLLOWING ACCEPTANCE OF YOUR PAPER:

• IMPORTANT NOTICE ABOUT OPEN ACCESS: To assist authors whose funding agencies mandate immediate public access to published research findings, The Journal of Physiology allows authors to pay an Open Access (OA) fee to have their papers made freely available immediately on publication.

The Corresponding Author will receive an email from Wiley with details on how to register or log in to Wiley Authors where you will be able to place an order.

• You can check if your funder or institution has a Wiley Open Access Account here:
https://authors.wiley.com/author-resources/Journal-Authors/open-access/author-compliance-tool.html

• TRANSPARENT PEER REVIEW POLICY: To improve the transparency of its peer review process, The Journal of Physiology publishes online as supporting information the peer review history of all articles accepted for publication. Readers will have access to decision letters, including Editors' comments and referee reports, for each version of the manuscript as well as any author responses to peer review comments. Referees can decide whether or not they wish to be named on the peer review history document.

• You can help your research get the attention it deserves! Check out Wiley's free Promotion Guide for best-practice recommendations for promoting your work at: www.wileyauthors.com/eeo/guide. You can learn more about Wiley Editing Services which offers professional video, design, and writing services to create shareable video abstracts, infographics, conference posters, lay summaries, and research news stories for your research at: www.wileyauthors.com/eeo/promotion.

• If you would like to receive our 'Research Roundup', a monthly newsletter highlighting the cutting-edge research published in The Physiological Society's family of journals (The Journal of Physiology, Experimental Physiology, Physiological Reports, The Journal of Nutritional Physiology and The Journal of Precision Medicine: Health and Disease), please click this link, fill in your name and email address and select 'Research Roundup':
https://www.physoc.org/journals-and-media/membernews

EDITOR COMMENTS

Reviewing Editor:

Thankyou again for your submission to The Journal of Physiology and for making significant amendments as per the reviewers comments. After a second round of review, both reviewers were in agreement that this work is acceptable for publication in The Journal of Physiology. Congratulations on completion of this large body of work, I look forward to seeing more from your group on this topic.

Senior Editor:

Thank you for the revised manuscript submission that has been considered by the original reviewing editor and reviewers. I am happy to convey that all were positive about the response to the reviewers comments and the revisions made by the authors. The manuscript was deemed acceptable for publication. Congratulations. Thank you for supporting The Journal of Physiology.

REFEREE COMMENTS

Referee #1:

Kia ora (hello). Thanks again for the opportunity to review this work. I appreciate the explanations offered by the authors in response to my queries. I remain of the view that the work is likely to be of interest to muscle, exercise, and metabolic physiologists, and that there is a rationale to justify the work in the context of recent studies investigating the same concept without untargeted proteomics.

The discussion is now suitably thoughtful and better highlights some of the limitations of the work.

Referee #2:

The authors have addressed my comments and edited the manuscript accordingly. I have no further comments.
